# Creating an arena for being in the same boat: A qualitative study of course instructors’ experiences with the Coping with Depression course’s group setting

**DOI:** 10.1371/journal.pone.0323545

**Published:** 2025-05-14

**Authors:** Eline Skraastad, Lise Sæstad Beyene

**Affiliations:** 1 Master of Addiction and Mental Health Work, Faculty of Health Science, University of Stavanger, Stavanger, Norway; 2 Faculty of Health Science, University of Stavanger, Kjell Arholms Gate 41, Stavanger, Norway; University of Education, PAKISTAN

## Abstract

**Background:**

Depression is a common mental disorder. Recovering from depression, a positive connection has been found between having a social network and having supportive relationships. Coping with Depression course was developed to address a need to reach more people through a non-stigmatising method of intervention outside the traditional mental health services. This course has proven effective in reducing depressive symptoms and is potentially cost-effective. Group dynamics can interfere with achieving a positive therapeutic effect, yet more knowledge is needed about the importance of the group setting of Coping with Depression courses.

**Aim:**

The study aims to gain a deeper understanding of the use of groups in the Coping with Depression course in primary health care.

**Method:**

A qualitative research design with thematic data analysis was applied to semi-structured interviews with seven course instructors.

**Results:**

Based on participant interaction, the findings indicate that the Coping with Depression course’s use of groups is predominantly beneficial. The group setting facilitated supportive fellowship and provided participants with an arena where social and personal growth was possible. Balancing the needs of individual participants with those of the group as a whole was challenging at times for the course instructors.

## Background

Depression is a common mental disorder affecting approximately five percent of the world’s population, with women more likely to be depressed than men [1]. Depression recurs for at least 50 percent of those who experience an initial episode, and the risk increases with each additional relapse [2]. Depression can create personal, social, and financial challenges for the individual and entail a socio-economic burden in the form of sickness absence and lost tax income, as well as increased social security and treatment costs [1,2]. However, it is estimated that around 75 percent of the population that develops a depressive disorder in low- or middle-income countries receives no treatment [1]. The treatment gap relates primarily to attitudes and cognitive barriers and secondarily to structural factors such as access, waiting lists, and finances [3]. Social networks and role expectations, as well as stigma and prejudice related to depression and mental health in general, can be obstacles to seeking help [3]. Social withdrawal and isolation are common among people with depression [4]. Nevertheless, with regard to living with and recovering from depression, a positive connection has been found between having a social network and having supportive relationships [4,5].

People with mild to moderate depression experience a reduction in depressive symptoms by participating in social group interventions [6]. The course in coping with depression, a cognitive behavioural group intervention based on social learning theory, was originally developed by Lewinsohn and colleagues and called the “Coping with Depression” (CWD) course [7]. The group typically consists of six to ten adults and meet weekly, with two follow-up sessions scheduled after one and six months [8]. The intervention, which was developed to address a need to reach more people through a non-stigmatising method of intervention outside the traditional mental health services [8], has been found effective in reducing depressive symptoms and potentially cost-effective [9]. The intervention is defined as a course in coping with depression, as it is intended to teach participants tools and coping techniques for dealing with depressive complaints and the symptoms expressed [8]. In contrast with traditional group therapy, patients are defined as course participants and the personnel conducting the course are known as course instructors [9]. The original CWD course program includes the teaching of social skills and behavioural activation, the identification of negative thoughts and problem-solving, mood monitoring, and relapse prevention [8]. Over the years, different versions of the CWD course have been developed and adapted to meet the needs of specific populations and intervention goals [9].

Research is lacking on experiences with the CWD course’s group setting. Existing knowledge about the group setting relates predominantly to intervention methods such as group therapy and counseling for people with depression and comorbid mental disorders [10–14]. Research shows that the majority find the group setting valuable in terms of normalisation as it makes it possible to meet others in similar situations [12,13]. When trust has been established, the group is experienced as a safe and supportive place. By sharing experiences and advice, participants can have an opportunity for new insight and growth [10–13]. On the other hand, group dynamics can interfere with achieving a positive therapeutic effect due to personal traits, such as shyness or dissatisfaction, or the presence of dominant participants [12]. More knowledge is needed about the importance of the group setting of CWD courses.

### Aim and research questions

This study aims to gain a deeper understanding of the use of groups in the CWD course in primary health care. The research question was as follows: what are CWD course instructors’ experiences with the group setting?

## Method

### Design

Qualitative design inspired by a phenomenological-hermeneutic approach [15] was chosen based on the research question’s articulation of a search for descriptive knowledge related to course instructors and their experiences with the group setting. This approach was suitable because it facilitated a thorough exploration of the course instructors’ personal experiences and perceptions within the group setting, providing a comprehensive understanding of the nuanced dynamics at play.

### Recruitment and sample

Recruitment was conducted by the first author through an online search to identify municipalities offering the CWD course. Informants were strategically selected [16] by contacting ten municipalities across various demographics and locations in Norway that offered the CWD course, providing them with information about the study and requesting their assistance in recruiting informants. The inclusion criterion for participation was experience as an instructor for a CWD course conducted between 2015 and 2022 to facilitate contemporary information and organised with physical attendance. A total of seven female informants from three different municipalities in the south-west and eastern part of Norway were recruited by email after providing consent. Five of these informants were recruited through contact persons at the municipality and two former course instructors were recruited by way of the “snowball method” [16]. The informants had two to eight years of CWD course experience. At the time of the interview, five were active course instructors and two were passive course instructors.

### Data collection

Seven individual in-depth interviews were conducted in December 2022 and January 2023. Six interviews were conducted at informants’ workplaces during working hours, and one interview was conducted digitally, on Zoom. Time spent ranged from 30 to 50 minutes. The interviews were conducted by the first author (ES) with the help of a semi-structured interview guide [16]. Open-ended questions were posed to obtain the informants’ experiences and opinions, including: “What experiences do you have with conducting CWD courses in groups for participants with depression?”, “How do course participants influence each other?”, “What are your thoughts on the impact of the group setting in a CWD course?”, and “Which factors do you consider affect the group’s cohesion?”. Follow-up questions were deemed appropriate for further clarification and elaboration. The interviews were audio recorded through the use of the Nettskjema-Diktafon app, a secure web-based tool for recording audio on smartphones, developed and hosted by the University of Oslo [17].

### Data analysis

Braun and Clarke’s [18] thematic analysis was used to analyse the data. The first author (ES) performed the first four phases of the analysis in collaboration with the last author (LSB), in which the text was systematised and categorised. In phase five, the interpretation was performed and validated by both authors. In the first phase, the first author familiarised herself with the data by listening to the audio recordings and then transcribing it verbatim, to ensure that the reproduction from speech to text corresponded with what the informants had communicated during the data collection. The text was then read through and keywords relevant to the research question were noted. In the second phase, the data material was transferred to NVivo to generate codes across the data material. In phase three, similarities and differences between the codes in the dataset were identified, which guided the sorting of the codes into relevant groups and their subsequent labelling. For example, the codes as “course participants understand each other,” “course participants support each other,” and “generosity and care in the group” were grouped under a preliminary category labelled “inclusion and emotional support.” In phase four, preliminary themes were developed based on interpretations of the data extracts associated with the codes. The category “inclusion and emotional support” was subsequently organized under the preliminary theme “social fellowship,” which, during the interpretation process, evolved into the theme “fostering a supportive group identity.” The themes were further systematized with sub-themes and were discarded if deemed irrelevant to the research question. The interpretation process moved back and forth between parts and whole in phase five, and the authors developed greater insight into and understanding of the data material, which led to the interpretation of the main theme [18] (Table 1).

**Table 1:** Overview of the results.

Overall theme	Creating an arena of being in the same boat		
Themes	Evolving group dynamics and interaction	Fostering a supportive group identity	Facilitating mutual learning and support within the group
Sub-themes	Balancing participation in the group Encouraging engagement from silent course participants	Creating a cohesive and supportive environment with a formal group structure Building social connections with an informal group structure	Sharing and contributing to mental growth Valuing peer insights and support

### Methodological considerations and limitations

The informants were recruited based on their experience as CWD course instructors within the primary healthcare service, and the study’s findings should be interpreted within this context [16]. Only female informants expressed interest in participating in the study. No notable differences were found between active and passive course instructors, or between the municipalities with regard to the data sample. Further nuances and enhanced generalizability of the study’s findings could have emerged had the sample size been larger, included both genders, and encompassed areas beyond the three municipalities Although an interview guide was used during data collection, the researcher’s interview technique [16] and formulation of questions may have influenced the informants’ answers. Although the interviews were audio recorded, dialect, mood, and pauses may have been misinterpreted during the transcription process. After the seventh interview, no new data relevant to the study emerged, and further recruitment was not considered necessary.

### Ethical considerations

This study was approved by Sikt – Norwegian Agency for Shared Services in Education and Research – and conducted according to ethical research principles as outlined in the international Helsinki Declaration [19]. The informants were informed orally and in writing about the aim of the study and guaranteed anonymity and confidentiality upon participation. Prior to the interviews, the informants were informed of the opportunity to withdraw from the study at any time, and consent to participate was obtained through their signature of a consent form.

### Findings

The study’s findings are described by the overall theme of “creating an arena of being in the same boat”. This overall theme was systematised with three themes and six sub-themes (Table 1).

#### Creating an arena of being in the same boat.

An overall finding of this study is that the informants experienced a sense of community and solidarity among their course participants. All involved cooperated and supported each other as they navigated common experiences and challenges, like being “in the same boat” during the CWD course. The group setting offered an arena that unified course participants who were in different life phases and had different characteristics and different experiences of depression. One informant described the course participants as being different but also the same. The informants experienced that the course participants developed a strong group affiliation relatively quickly. They conveyed that the group setting helped to facilitate giving course participants insight into the experiences of others and an experience of not being alone in the world with the challenges triggered by depression.

Being in the same boat was the red thread throughout the data material. The three themes represent the informants’ various experiences of the group setting of CWD courses.

#### Evolving group dynamics and interaction.

The informants experienced differences in interaction and dynamics in various groups. Some groups were more active and others more passive. During the start-up phase of the courses, it was experienced that the group atmosphere was tense, and course participants were hesitant and cautious about speaking. One informant described the start as “the first day of school”, where course participants related more to the course instructors than to each other. As the course participants got to know and trust each other, interaction and dialogue in the group increased.

*Balancing participation in the group*: There were positive experiences throughout with extroverted course participants who were bold and dared to speak up in the group. The active course participant sets an example for the rest of the group, encouraging other course participants to come forward in the dialogue, and was described as a person who positively took responsibility and initiative for interaction in the group. An informant described the experience as follows:

*Those who are the boldest and talk the most set the standard in a way. And then the others dare to come forward themselves. Then we see that when one or two come forward, for example, in the presentation and say “I’m here because I’m so depressed, everything has been shit”, that it tells [something] a little personally. Then we see that people share their things much more easily. They (…) are just like a door opener for the rest of the group.* (Participant No. 2)

On the other hand, there was a balance to be struck between letting the active course participant have space and not preventing other course participants from providing their own input. Several informants found that individual course participants had difficulty limiting themselves. In this case, the informants tried to calm the active course participants positively so that they did not feel offended. Techniques the course instructors used in such situations included putting the course participants in smaller groups or asking the group open-ended questions. It was necessary to continually assess how much time and space active course participants should have, and the challenge was to balance this against taking care of the quiet course participants and ensuring that they too were included in the dialogue.

*Encouraging engagement from silent course participants*: Several informants said that taking care of the silent course participants who rarely spoke was a more common problem in groups than having to suppress the active ones. The informants conveyed that they had to take control of the dialogue to a greater extent to ensure that the silent course participants also had the opportunity to participate and could, for example, be facilitated by going around the table. To prevent them from disconnecting, the informants conveyed that they asked questions, they assumed the course participants could relate to. One informant described trying to facilitate the inclusion of the silent course participants:

*Perhaps try to engage them and involve them with small drops. In a way, don’t demand too much so that it becomes an uncomfortable setting if they don’t want to be so visible, but also don’t let them disappear completely. But make sure, perhaps by small simple questions along the way, that that person is connected and following along. So, just by asking because then, in a way, we give a signal that we see you and there is room for you too.* (Participant No. 5)

The informants reported that taking care of the silent course participants became a challenge they tried to address by involving them in the group dialogue and giving them space. The informants emphasized that there was no requirement to say anything out loud or answer questions. However, they encouraged participation hoping for increased verbal engagement as the course participants became more secure in the group. The balance was to get the silent actively involved in the dialogue without this being perceived as burdensome or unsafe for the individual course participant.

#### Fostering a supportive group identity.

It was the informants’ experience that the groups became a “we” and a good place to be. The atmosphere created an impression of acknowledgment and support and an experience of wishing each other well. Within this theme, two sub-themes were identified based on the formal group structure and the informal group structure.

*Creating a cohesive and supportive environment with a formal group structure*: Although the course participants differed in age and gender and had different life experiences, it was the experience of all informants that the group became an arena that brought course participants together. The group developed a sense of fellowship, where interaction increased as they got to know each other better. Several had experiences with course participants who described the group as useful because of the opportunity to meet like-minded people who understood what it was like to live with depression. The informants experienced that acknowledgment and understanding of one another’s life experiences, care for and generosity towards each other, and a genuine interest in wishing each other well were all factors that bound the course participants together. An informant shared the following experience:

*That’s what a lot of people say when they join the group: “It’s so good to meet others who are in the same situation, who understand. You don’t have to explain so much because in a way they have been there themselves.”* (Participant No. 7)

*Building social connections with an informal group structure*: This sub-theme deals with the informal processes that take place between the course participants outside the organised group structure. Each course session included breaks when the course instructors left the teaching room. The breaks were the course participants’ arena, where they could talk about whatever they wanted and freely share topics they probably would not have been comfortable with in the formal group structure. As an informant said:

*We [course instructors] always go out during the break and they [course participants] (…) always find it uncomfortable at first. But now we hear them from a long way off. And when we return: “Oh no, are you here already? We’re not done yet!” (…) Now there is a Netflix series they have discussed.* (Participant No. 1)

The social fellowship in the groups was described as a positive additional effect of the group setting. It allowed the course participants to expand their social network by establishing new relationships that offered the potential for support both during and after the CWD course. On the other hand, it was up to the individual course participant to choose to take part in the fellowship, and some informants described sporadic experiences, with course participants who did not connect and largely kept to themselves.

#### Facilitating mutual learning and support within the group.

The third theme was the group setting’s contribution to course participants having the opportunity to learn from each other. Several informants found the interaction between course participants to be probably as important as the course instructors’ teaching. Course participants being gathered into a group where they could make use of each other’s experiences was described as a positive therapeutic effect. The informants indicated the value and usefulness of course participants being able to both share and receive.

*Sharing and contributing to mental growth*: This sub-theme refers to course participants’ ability to give something back to the group, either by sharing experiences or by asking questions and giving advice and tips. The course participants were encouraged to share their own experiences in the group, as this could give them new insight into their thought and behaviour patterns, as well as an opportunity to feel what coping is like by daring to say this out loud. In addition to the personal benefit, the informants experienced a social gain from the group being able to provide feedback and response, and from the opportunity to make use of each other’s experiences. One informant described an episode in which she realised the value of sharing with the group which meant that she learned something new:

*We talked about what you liked to do before, you should start doing again. One person liked paddling in a kayak but had withdrawn from the kayaking and become more inactive. So, yeah, that was going to be his goal, and he started paddling. He shared with the group that he didn’t feel joy right away. He had to repeat the paddling a few times (…) to make his body remember that this was “I like to do this”, then the joy comes.* (Participant No. 6)

From the informants’ experiences, they found that when course participants expressed their thoughts and feelings, it allowed them to ask reflective questions. They reported a key responsibility to transform individual experiences into broader topics, enabling the entire group to engage in discussions and learn collectively.

### Valuing peer insights and support

Although the CWD course educates course participants about depression, as well as providing tools and coping techniques for use in everyday life, several informants said that being able to listen to and accept the experiences of other course participants seemed to be just as important and valuable. One informant described input from the course participants as being worth its weight in gold. Advice and tips from people who understand what it is like to have and live with depression can be perceived as more credible. At the same time, experiences were often recognisable, which confirmed for the course participants that the thoughts they had were not a consequence of being “crazy”. One informant shared the following experience:

*A young man (...) who was so reflective shared his experiences. Some of the mature ladies said “This was so helpful and so good! This inspired me!”* (Participant No. 4)

The informants found that it was important to arrange for the course participants to be able to receive input about others’ experiences through open discussion together. It gave the course participants insight into other ways of thinking and acting and an experience of not being different or alone with their challenges.

## Discussion

The aim of this study was to gain a deeper understanding of the use of groups in the CWD course in primary health care. The research question addressed CWD course instructors’ experiences with the group setting. The course instructors’ experience of the group was that it created an arena for the course participants to be in the same boat, and this included the themes of evolving group dynamics and interaction, fostering a supportive group identity, and facilitating mutual learning and support within the group.

This study shows that interaction between the participants was a valuable and important supplement to the group setting of the CWD courses. The course instructors wanted the participants to have active dialogue, which they facilitated by asking open questions for the purposes of reflection and sharing. The course instructors had to strike a balance in order to regulate group dialogue such that all participants could participate in their own way according to their individual preferences, and challenges could arise in the meeting between active and silent participants. On the other hand, there is a risk that the presence of the course instructors could obstruct active dialogue in that the individual expertise of the participants is overshadowed by the presence of the professional [20]. In general, the indication is that the more active and influential a participant is within a group, the more likely that person is to benefit from the group [20]. Each group member will influence the social climate through their behaviour [20]. Sharing private experiences can build trust within a group, and active participants who reveal their thoughts and feelings act as contributors to the group dialogue [13]. If the reactions of and feedback from other participants are based on respect and care, then a safe and reliable group atmosphere is created, which helps more people dare to share freely [13,20]. On the other hand, the group setting can become a barrier to equal participation by participants as the result of different personality characteristics [12], which corresponds to findings from this study.

Even though some participants were not verbally involved, it was the informants’ experience that the silent participants participated in the active dialogue through affirming body language and active listening. Nevertheless, silent participants can be just as big a challenge in group dynamics as active participants [20]. Self-disclosure is not only crucial for the development of group cohesion, but it also correlates directly to positive therapeutic effects. Increased verbal participation contributes to increased commitment and appreciation from other participants [20]. There are several possible reasons for a participant’s silence. It is conceivable that symptoms related to depression create a barrier to verbal participation. Fear and anxiety, lack of interest, and hopelessness are described by people with depression as constraints to participation in leisure activities [21].

Passivity and withdrawal from social settings are common symptoms of depression [2]. This study found that the group setting contributed to the development of a social community, regardless of group composition in terms of age and gender. The formal group structure, in which participants worked together towards a common goal, created a sense of belonging. Intervention methods that contribute to increased social contact and by means of which participants identify with the group have been found to reduce depressive symptoms over time [22]. Groups with strong cohesion show a higher degree of satisfaction, attendance, participation, and mutual support [20]. This may have contributed to the fact that participants made new acquaintances and relationships through the informal structure formed during the breaks, as identified by this study. On the other hand, informal group structures can contribute to the formation of subgroups within groups, with participants being included or excluded. Such subgroups can have consequences for group fellowship and complicate unity by creating polarisation or marginalisation [20].

The study finds a synergy effect in groups where participants can take on two roles, as both helper and receiver. Although the group setting is associated with a CWD course, the informants point out that the interaction and learning that take place between the informants are just as important. This is in line with the principle of self-help groups, where the therapeutic effect is expressed through the exchange of experience. Participating in self-help groups can give one hope and the motivation to change one’s life situation [23]. Gathering participants with similar challenges in a group creates an arena that offers the potential for learning and growth and can be related to observational and model learning [20]. By observing, one can learn behaviour. On the other hand, for behaviour to be learned, it is a prerequisite that it be understood, remembered, and applied appropriately in one’s own life [24]. By receiving, one can create distance from one’s challenges and gain new insight from the observation of the alternative coping techniques of others. At the same time, being able to be the one who gives can help mobilise personal effort and provide greater insight into one’s resources, which one can use to help oneself [20,23]. This is in line with the study’s findings and with previous studies which show that participants can act as role models when offered new and more appropriate behaviour and thought patterns [10].

## Conclusion

The aim of this study was to gain a deeper understanding of the use of groups in the CWD course in primary health care. The findings show that the group setting develops a supportive fellowship and becomes an arena that allows opportunities for personal and social growth. The group setting creates an interaction effect, whereby participants can learn from each other through the observation and exchange of experiences, as well as an opportunity to form new relationships. The findings indicate challenges in facilitating active dialogue within the groups due to different personalities and commitments. Active course participants are found to be a resource in the group that creates greater commitment and interaction between participants, but which can also obstruct others’ input if allowed to take up too much space. It is a balancing act to include both the active and the silent course participants in the group while respecting the participants’ individual preferences and needs.

The study is believed to have elicited experiences related to the research question, with these findings considered representative of and useful for other CWD course instructors. It remains challenging to determine whether the observed findings are specifically attributable to the type of treatment (group vs. individual), the nature of the treatment (CWD course vs. other interventions), or the characteristics of depressed persons. Further research is needed to disentangle these factors and provide clearer insights into the effectiveness of different treatment modalities and their impact on diverse populations. Greater knowledge about and understanding of the group setting can stimulate greater use of mental health care in groups for people with depression. In this way, the primary health service can reach more users with necessary and knowledge-based mental health care in a cost-effective manner. However, there is also a need for a broader understanding of the group setting from the course participants perspectives. Further research should examine the experiences of course participants who have completed a CWD course in a group and the utility value of the CWD course as compared to individual interventions for people with mild to moderate depression.
