# Correction: Creating an arena for being in the same boat: A qualitative study of course instructors’ experiences with the Coping with Depression course’s group setting

**DOI:** 10.1371/journal.pone.0334054

**Published:** 2025-10-08

**Authors:** The PLOS One Staff

There is an error in reference 17. The correct reference is: University of Oslo. Nettskjema: University of Oslo; Available from: https://nettskjema.no/?lang=en.

The publisher apologizes for the error.
